# Developing and Validating a Childhood Trauma-Informed Curriculum for Primary School Teachers in Limpopo Province, South Africa

**DOI:** 10.3390/children12091256

**Published:** 2025-09-18

**Authors:** Muimeleli Munyadziwa, Lufuno Makhado

**Affiliations:** Department of Public Health, Faculty of Health Sciences, University of Venda, Thohoyandou 0950, South Africa; lufuno.makhado@univen.ac.za

**Keywords:** childhood trauma, Limpopo province, primary school teachers, trauma-exposed learners, trauma-informed curriculum

## Abstract

**Highlights:**

**What are the main findings?**

- Primary school teachers in rural Limpopo have limited knowledge and training on childhood trauma, affecting their ability to support trauma-exposed learners.
- The developed trauma-informed curriculum addresses gaps in awareness, early identification of trauma, and classroom strategies to create supportive learning environments.

**What is the implication of the main finding?**

- Implementing the curriculum can empower teachers to respond effectively to trauma-exposed learners, improving educational and psychosocial outcomes.
- The curriculum provides a model for trauma-informed teacher training in under-resourced schools, with potential applicability in similar contexts globally.

**Abstract:**

**Background/Objectives**: Childhood trauma significantly hinders the developmental and academic outcomes of learners, particularly in under-resourced schools such as those in Limpopo province, South Africa. Teachers in these settings often face challenges in supporting trauma-exposed learners due to a lack of knowledge, training, and appropriate resources. Addressing this gap requires the development of structured, trauma-informed educational support systems. **Methods**: This study forms the final phase of a multi-phase research project aimed at developing a trauma-informed curriculum for primary school teachers. A multi-phase mixed method design was adopted across four phases: (1) a global scoping review to identify effective trauma-informed interventions; (2) empirical interviews with primary school teachers, trauma center managers, clinical psychologists, and social workers to understand local needs and experiences; (3) development of a conceptual framework grounded in theoretical and empirical findings; and (4) curriculum development guided by El Sawi’s curriculum design model. The curriculum was validated using structured questionnaires with a panel of stakeholders including educators, mental health professionals, and curriculum experts. **Results**: The study identified critical issues, including teachers’ limited understanding of childhood trauma, lack of standardized training, and inadequate classroom strategies. Key curriculum components were developed to address these gaps, including modules on the nature of trauma, early identification of symptoms, trauma-informed teaching practices, and collaboration with mental health professionals. Validation results indicated strong agreement on the curriculum’s clarity, relevance, and potential impact. **Conclusions**: The developed trauma-informed curriculum provides primary school teachers in Limpopo with the knowledge, tools, and confidence to support trauma-exposed learners. It emphasizes early identification, responsive classroom strategies, and inter-professional collaboration. This curriculum has the potential to enhance learning environments and promote better educational and psychosocial outcomes for trauma-affected learners.

## 1. Introduction

Childhood trauma is a widespread problem that has severe and long-term consequences for children’s psychological, emotional, and academic development [1,2,3,4,5,6]. Teachers in primary schools are frequently on the front lines, watching how trauma impacts children’s behavioral and academic issues. However, numerous teachers lack the knowledge and resources to appropriately respond to trauma cases [7,8,9]. This statistic is obvious in Limpopo Province, where socioeconomic and community-based pressures worsen trauma exposure. Poverty, marital violence, substance addiction, and communal violence are all substantial contributors to bad childhood experiences, putting children at risk for developmental delays. Childhood trauma is becoming more widely recognized as a public health concern with substantial educational effects [10,11].

In response to this, countries like the United States (U.S.) and the United Kingdom (UK) have initiated trauma-informed education policies that encourage schools to integrate trauma-sensitive practices into their teaching model [12,13]. Such practices include creating safe and predictable environments, building learners trust, and teaching emotional regulation skills [14]. These approaches have positively affected student engagement and academic performance, particularly among at-risk populations [15,16]. The effectiveness of trauma-informed training for educators has been well-documented across various studies. Research shows that when teachers are trained to understand the impacts of trauma, they are better equipped to respond with empathy and create supportive learning environments [17,18,19].

However, while these policies have achieved success in the U.S. and U.K., their direct applicability to South Africa, particularly in resource-constrained and rural settings such as Limpopo, requires careful consideration. The South African education system is influenced by unique historical, cultural, and socioeconomic factors that differ from those in the Global North. Limited resources, large class sizes, and inadequate access to psychosocial support services make it challenging to directly implement Western models. This necessitates adapting these frameworks to fit the local context, integrating community-based perspectives and culturally relevant practices [20,21]. This adaptation aligns with ongoing debates on the decolonization of the curriculum, which argue for the development of educational approaches that reflect South Africa’s lived realities and historical context [20].

Childhood trauma in South African schools is also deeply intertwined with trauma in surrounding communities [22]. The legacy of apartheid continues to shape systemic and structural inequalities, with poverty, unemployment, violence, and social instability remaining prevalent in many communities [23]. These factors not only expose children to trauma but also normalize it within their social environments, leading to complex behavioral and emotional challenges in schools. School violence predominantly affects learners and can be carried out by fellow students, educators, or other members of the school community, further compounding the challenges of creating safe and supportive learning environments [22,24]. The historical and structural violence rooted in apartheid-era policies perpetuates patterns of aggression, mistrust, and fear that continue to play out in contemporary educational settings. Despite growing recognition of trauma-informed education, little research explores how it can be adapted to rural South African contexts, leaving gaps in strategies for teachers and learners in Limpopo.

This study is grounded in a combined theoretical framework based on SAMHSA’s trauma-informed model and a transformative approach. SAMHSA identifies key principles (realize, recognize, respond, and resist retraumatization) that inform trauma-informed practices [25]. The transformative lens emphasizes empowerment, cultural relevance, and stakeholder collaboration, ensuring curriculum development fosters systemic change rather than superficial interventions [26]. In line with this, teachers who undergo trauma-informed training report feeling more confident in managing classroom challenges and are more likely to utilize strategies that prevent re-traumatization [27,28].” Furthermore, trauma-informed training programs have been linked to improvements in teacher well-being and retention, as teachers experience less stress and burnout when they feel equipped to handle the emotional needs of their students [29]. Research indicates that when teachers are unprepared to manage trauma-related behaviors, it can negatively affect the classroom environment, leading to disruptions in learning and a sense of insecurity among students [30,31].

Yet, evidence also highlights the transformative role that trauma-informed educational practices can play in supporting children’s recovery and resilience [32]. Given the urgency of these issues in high-risk regions like Limpopo, it is imperative to develop contextually relevant, culturally sensitive, and resource-conscious training programs for educators. This study therefore aims to develop a trauma-informed curriculum tailored for primary school teachers in Limpopo province. By equipping educators with the tools and knowledge to address childhood trauma, the initiative seeks to cultivate more supportive learning environments and improve outcomes for trauma-affected learners.

**Aim:** The aim of the study is to develop and validate a trauma-informed curriculum for primary school teachers in Limpopo province, South Africa, that equips educators with the knowledge and practical strategies to identify, respond to, and support learners affected by childhood trauma.

## 2. Materials and Methods

This study is part of the fourth and final phase of a broader research project to develop a trauma-informed curriculum for primary school teachers in Limpopo province (LP), South Africa. The main goal is to reduce the impact of trauma on primary school learners by equipping teachers with a thorough understanding of childhood trauma and effective strategies to address it. The research utilized a multi-phased mixed method approach organized into four distinct phases. Phase one entailed conducting a scoping review to investigate the effectiveness of trauma-informed interventions across international contexts.

### 2.1. Study Population and Sampling (Development)

The study was conducted in the Vhembe district of Limpopo province, South Africa, focusing on the Mvudi and Dzindi circuits. A total of twenty-five (25) primary schools were included, comprising thirteen (13) schools from Mvudi and twelve (12) from Dzindi. Participants were selected purposively based on their experience and relevance to childhood trauma and education.

Inclusion criteria required participants to be over eighteen (18) years old and have at least five (5) years of experience in child development or trauma-related work, while non-teaching staff and professionals outside the study area were excluded.

During the curriculum development phase, a multidisciplinary team of eleven (11) members guided the design process to ensure the curriculum was comprehensive and contextually appropriate. This team consisted of three (3) researchers, two (2) qualified curriculum advisors, two (2) clinical psychologists, two (2) social workers, and one (1) trauma center manager.

### 2.2. Steps to Developing a Childhood Trauma Curriculum

**Figure 1:**
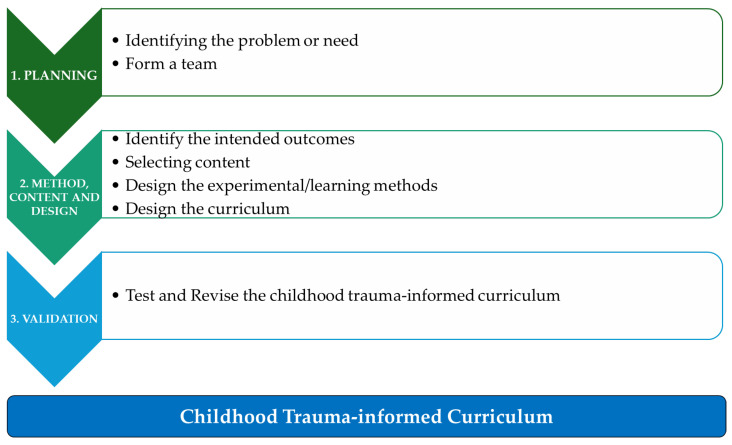
Essential steps to developing a childhood trauma-informed curriculum.

The researchers adopted and moderated the Guide: Population Education for Non-formal Education programs of out-of-school Rural youth [33], as shown in Figure 1. The adopted guide consists of four (4) steps; in the current study, the researchers have three (3) steps that were followed to develop the childhood trauma-informed curriculum. The initial step will be planning, which consists of identifying the problem or need and forming a development team. This will be followed by methods and content, including identifying the intended outcomes, selecting content, and designing experimental methods. Step 3 is validation, where the developed childhood trauma curriculum will be tested and revised.

**Step 1: Planning**

**a.**  **Identify the issue/problem/need through needs assessment and analysis.**

The problem was identified from phases 1 and 2 and several recommendations emerged; actions to fulfill the recommendations were to be taken to ensure that the trauma-informed curriculum is developed. The scoping review, which applied the Arksey and O’Malley framework [34], has produced several important problems as identified in the results section.

**b.**  **Form curriculum development team**

In this step, the researcher had a team of about Eleven (11) members with different roles and functions.

Three (3) Researchers, Two (2) Qualified curriculum advisors, Two (2) Clinical psychologists, Two (2) Social workers and One (1) trauma center manager

**Step 2: Methods, Content and Design**

**a.**  **State intended outcomes.**

In this research study, the intended outcome was already established, which was that primary school teachers would be trauma-informed and able to apply theory into practice.

**b.**  **Select content.**

The content for the trauma-informed curriculum was selected by aligning it with the recommendations derived from Phase 1 (scoping review) and Phase 2 (empirical phase). The goal was to address the identified gaps and challenges that teacher face in identifying and supporting trauma-exposed children. The recommendations from both phases pointed to several key areas that guided the development of the curriculum content.

**c.**  **Design experimental methods**

In this step, the researchers planned activities that enabled the class students to achieve the desired results. This incorporates learning styles and a list of the activities that had to be completed. A worksheet for the facilitators and brief discussions of the lesson delivery method and the learning environment.

**d.**  **Design**

In this step, the researchers finalized the curriculum design as shown in Table 1.

### 2.3. Data Collection Method (Development)

Findings from Phase 1 (scoping review) and Phase 2 (empirical phase) identified gaps and guided the content and structure of the curriculum. Semi-structured interviews were conducted during the empirical phase to gather in-depth insights from teachers and mental health professionals about challenges, needs, and culturally sensitive practices.

### 2.4. Data Analysis (Development)

Data from Phase 1 (scoping review) and Phase 2 (empirical research) were analyzed to identify key themes, gaps, and recommendations for the curriculum. Scoping review findings were organized to highlight existing knowledge, evidence-based strategies, and teacher training gaps. Interview and focus group data were transcribed and analyzed using thematic analysis, generating themes on teacher experiences, challenges, and training needs. Findings from both phases were synthesized to determine priority curriculum areas, including trauma definitions, early signs, classroom strategies, teacher self-care, and school-based support. Themes were iteratively reviewed and refined by the development team to ensure content was contextually relevant, practical, and aligned with identified needs.

### 2.5. Validation Method (Step 3)

In this phase, the researchers tested and refined the developed Childhood Trauma-Informed Curriculum, as illustrated in Table 1. All relevant stakeholders, including teachers, mental health professionals, and curriculum specialists, actively participated in both the design and validation process. Their input was essential to ensure that the curriculum accurately reflected their needs, was feasible for implementation in real school settings, and was clear, user-friendly, and practical.

### 2.6. Study Population and Sampling (Validation)

During the validation phase, thirty (30) individuals were invited to participate, and twenty-six (26) consented. A purposive sampling strategy was used. To be eligible, participants had to be older than eighteen (18) years and possess at least five (5) years of experience in child development or trauma-related work. Individuals who were non-teaching staff or professionals outside the study area were excluded. The research took place in Limpopo Province, where many children are affected by trauma linked to poverty, violence, sexual abuse, and other socio-economic difficulties.

### 2.7. Data Collection (Validation)

To collect stakeholder feedback, a questionnaire was used. This allowed participants to provide rich, descriptive feedback that complemented the multimethod design of the study, enhancing the credibility and depth of the validation process.

The questionnaire was structured into five sections: (A) Clarity and content relevance, (B) Teaching methods and strategies, (C) Practical application and usability, (D) Overall impression, and (E) Additional comments. Open-ended questions in each section invited participants to reflect on the curriculum’s relevance, clarity, applicability, and feasibility in a rural South African context, and to suggest improvements or additional content.

The feedback obtained through the questionnaire, together with collaborative discussions with stakeholders, informed iterative revisions of the curriculum. This process ensured that the final product was contextually appropriate, culturally sensitive, and practical for use in primary schools in Limpopo province.

### 2.8. Data Analysis (Validation)

Data from the validation questionnaire were analyzed using descriptive statistics to capture the extent of agreement across different aspects of the curriculum. Responses to open-ended questions across all sections (Clarity and Content Relevance, Teaching Methods and Strategies, Practical Application and Usability, Overall Impression, and Additional Comments) were analyzed. Participants evaluated the curriculum using structured questionnaires with Likert-scale items and open-ended questions, providing both quantitative ratings and qualitative feedback. Feedback was organized into four main areas: clarity and content relevance, teaching methods and strategies, practical application and usability, and overall impression. Percentages were calculated for each item to reflect the level of consensus among respondents. This approach made it possible to identify not only the strengths of the curriculum but also areas that may require refinement, providing a balanced view of its overall suitability for use in educational settings.

## 3. Results

During the validation phase, the researcher assembled a multidisciplinary team of eleven (11) members with diverse roles to guide the curriculum design process. The team comprised:

- Three (3) Researchers
- Two (2) Qualified curriculum advisors
- Two (2) Clinical psychologists
- Two (2) Social workers and
- One (1) trauma center manager

Participants were recruited physically by the researcher, who visited schools, Department of Education offices, and community/trauma centers to explain the study and obtain signed consent forms.

During the validation phase, thirty (30) individuals were invited, of whom twenty-six (26) consented to participate. The team included three (3) clinical psychologists, three (3) curriculum advisors, nine (9) primary school teachers, four (4) researchers, and nine (9) social workers. Participants were recruited using multiple methods, including sending the questionnaire via WhatsApp and email, as well as in-person visits to schools, clinics, and Department of Education offices where consent forms were provided. Among the participants, fourteen (14) were female and twelve (12) were male. Ages ranged from twenty (20) to twenty-nine (29) years (*n* = 2) and thirty (30) to thirty-nine (39) years (*n* = 14), as presented in Table 2.

Identify the issue/problem/need through needs assessment and analysis.

The problem was identified from phases 1 and 2 that were conducted, and several recommendations emerged, and actions to fulfill the recommendation were to be taken to ensure that the trauma-informed curriculum is developed. The scoping review, which applied the Arksey and O’Malley framework [26] has produced several important themes as identified.

### 3.1. Findings from Phase 1 (Scoping Review)

The scoping review identified several key areas highlighting gaps in teachers’ knowledge and school preparedness to address childhood trauma. Three main themes emerged:

#### 3.1.1. Theme 1: Insufficient Teacher Knowledge and Training

Teachers often lacked comprehensive knowledge about childhood trauma and its impact on learning. Evidence from reviewed studies suggested that trauma-informed practices were rarely integrated into teacher training programs. The scoping review found that teachers’ knowledge of trauma strongly affects the success of trauma-informed interventions. Adequate training enables teachers to better recognize and respond to trauma, while comprehensive programs improve their ability to apply supportive strategies [35,36,37,38].

- Recommendation: Teachers should have a clearer, standardized definition of childhood trauma, and an understanding of the different forms trauma can take, such as physical, emotional, sexual abuse, and neglect. Furthermore, Teachers need guidance on recognizing the early signs and symptoms of trauma, including behavioral, emotional, and academic indicators.
- Curriculum Content: The curriculum includes a module dedicated to defining childhood trauma, highlighting various forms and effects of trauma (e.g., abuse, neglect, witnessing violence). This ensures that teachers have a consistent and comprehensive understanding of the concept, which is essential for identifying trauma-exposed students early. It is also vital that the curriculum incorporates a module focused on recognizing the early signs of trauma, emphasizing behavioral indicators (e.g., aggression, withdrawal), emotional indicators (e.g., crying, anxiety), and changes in academic performance (e.g., decline in grades or interest in school). Teachers are trained in how to identify these signs in their students to facilitate early intervention.

#### 3.1.2. Theme 2: Enhancing Teacher Well-Being and Professional Capacity

The review highlighted that trauma-informed programs not only benefit learners but also enhance teacher well-being, reduce burnout, and improve self-regulation practices [37,39]. The scoping review recommends that teacher training should include structured modules on self-regulation and wellness, alongside ongoing professional development opportunities. These programs should equip teachers with strategies to manage stress, prevent burnout, and maintain resilience in high-stress environments.

- Recommendation: Teachers may experience secondary trauma and burnout from dealing with traumatized students, so self-care routines are essential.
- Curriculum Content: A module on teachers’ self-care techniques was included to help educators manage their own well-being, preventing burnout and secondary trauma. The module covers techniques such as mindfulness, setting boundaries, and seeking peer support.

#### 3.1.3. Theme 3: Strengthening Learner Resilience, Relationships, and Academic Engagement

Findings from the scoping review revealed that trauma-informed curricula in schools should integrate social-emotional learning, resilience-building activities, and relational practices. Such approaches should prioritize creating safe and trusting environments where learners can actively participate, build coping strategies, and achieve improved academic outcomes [40,41,42,43]

- Recommendation: There should be specific strategies for teachers to create a trauma-sensitive classroom environment, addressing the psychological and emotional needs of trauma-exposed children.
- Curriculum Content: A module on classroom trauma-informed strategies was developed, where teachers are trained in creating a safe, supportive learning space. This includes strategies such as providing structure, offering emotional support, and ensuring consistency in expectations, which are known to help trauma-affected students feel secure and succeed academically.

### 3.2. Findings from Phase 2 (Empirical Phase)

While issues that were identified from phase 1, more problems/themes identified from phase 2 (Empirical phase) also contributed to the development. Here are the themes identified from Phase 2, based on the findings as well as the recommendations and curriculum content.

#### 3.2.1. Theme 1: Standardizing the Definition of Trauma [44]

- Recommendation: Teachers were found to have different interpretations of what constitutes childhood trauma. A unified, clear definition is essential for consistency. Some narratives from participants are as follows:

*“I think child trauma is when a child is having problems at home or even at school.”*

(Participant MA)

*“Child trauma…can be physical abuse, emotional abuse or sexual abuse.”*

(Participant MB)

- Curriculum Content: The curriculum starts with a comprehensive definition of childhood trauma, aligning it with formal definitions like those from SAMHSA, and integrating real-life examples. This ensures teachers understand trauma in its various forms and how it affects children’s behavior and academic performance.

#### 3.2.2. Theme 2: Identifying At-Risk Learners Through Behavioral, Emotional, and Academic Indicators

- Recommendation: Teachers should be trained to identify trauma through behavioral changes, emotional expression, and academic performance. Some narratives from participants are as follows which highlights how primary school teachers said they would identify a child with trauma:

*“You can identify him/her just because he cannot concentrate…on what I am doing in the classroom.”*

(Participant MA)

*“Sometimes they’d just be so quiet in class. Then you can see that this one is not here in class.”*

(Participant MB)

- Curriculum Content: The module on recognizing early signs of childhood trauma emphasizes all types of indicators: from changes in behavior (e.g., aggression, withdrawal) to emotional responses (e.g., sadness, fear) and academic performance (e.g., drops in grades). Instructors are also encouraged to look for more subtle symptoms, such as physical complaints (headaches, stomachaches), and internalized stress.

#### 3.2.3. Theme 3: Need for Adequate School-Based Support Systems

- Recommendation: Schools need in-house mental health professionals or support systems to assist with trauma cases rather than relying solely on external resources. Insights from participants included:

*“If we could have an office for nurses where children can go and explain their struggles, it will help.”*

(Participant MF)

*“We once suggested that sometimes we can get a psychological social worker.”*

(Participant MB)

- Curriculum Content: While the curriculum is primarily focused on teaching trauma-informed strategies, the support systems module highlights the importance of collaboration with healthcare professionals and community resources. Teachers are encouraged to establish a network of support within the school, and leadership training is recommended for school principals and heads of department to better manage cases of trauma.

#### 3.2.4. Theme 4: Addressing Teacher Knowledge Gaps and Providing Ongoing Professional Development

- Recommendation: Many teachers lack the necessary training and ongoing professional development to handle trauma-related issues effectively. Teachers described their experiences as follows:

*“There is nothing in our curriculum; we have never been trained on child trauma.”*

(Participant MC)

*“I would attend a childhood trauma workshop; it would really help us.”*

(Participant MI)

- Curriculum Content: The curriculum is designed to be an ongoing professional development tool. It includes both theoretical and practical approaches, such as role-playing and case study analysis, to ensure that teachers not only learn but also practice and internalize trauma-informed approaches. There is also an emphasis on continuous teacher reflection and peer support, ensuring that trauma-informed practices are embedded within the teaching profession.

In the final step, the researchers finalized the curriculum design as shown in Table 1.

#### 3.2.5. Validation Findings

The results show how participants viewed the curriculum’s effectiveness in addressing childhood trauma, how practical it would be to implement in schools, and how well it could help teachers support learners who have experienced trauma. Table 3 summarizes the evaluation scores and percentage of participants agreeing with each item.

#### 3.2.6. Clarity and Content Relevance

Most participants rated the curriculum as highly relevant to addressing early childhood trauma in educational settings. Specifically, 92% of respondents indicated that the curriculum effectively covered essential aspects of early trauma, while 100% agreed that the learning objectives were clearly articulated and achievable. Most participants (96%) found that the content of each module addressed the needs of primary school teachers, and 58% felt that no additional topics were necessary. Themes from open-ended responses included the curriculum’s comprehensive approach and practical classroom strategies. Social workers (3) and psychologists (3) emphasized the importance of including mechanisms for reporting abuse and providing specialized interventions. Primary school teachers (5) highlighted practical strategies for classroom management, while curriculum advisors (2) and researchers (3) focused on clarity, feasibility, and teaching methodologies. The high level of agreement may reflect the participants’ prior familiarity with these teaching methods or their recognition of evidence-based practices in trauma education.

#### 3.2.7. Teaching Methods and Strategies

Most participants found the recommended teaching approaches appropriate and effective, with 96% agreeing that methods such as seminars and role-playing adequately engaged teachers, and 100% stating that these methods helped teachers understand and apply trauma-informed practices. Around 77% did not suggest any alternative teaching methods. Open-ended responses highlighted suggestions for mentoring, individualized coaching, and the use of simulations to reinforce learning. Social workers (2) recommended ongoing workshops and one-on-one coaching, while psychologists (2) suggested practical demonstrations for better understanding. Female participants (8) frequently highlighted learner-centered strategies, whereas male participants (4) focused on logistical feasibility.

#### 3.2.8. Practical Application and Usability

Participants largely considered the curriculum feasible to implement in real school settings, with 96% rating implementation as practical. Approximately 81% believed that available resources and materials were sufficient to support learning, and 96% agreed that module durations were appropriate. A minority (16%) noted potential logistical challenges, such as resource availability or the need for additional training workshops. Qualitative themes included the ease of integrating the curriculum into daily classroom practice, the importance of clear instructions, and the need for supplemental workshops. Experienced teachers (4) focused on practical applicability, while newer teachers (3) emphasized clarity and usefulness of teaching methods.

#### 3.2.9. Overall Impression

The curriculum received an overall positive evaluation, with 96% of participants rating it as excellent. Most participants (77%) did not have additional suggestions for improvement. Comments indicated that the curriculum would enhance teachers’ ability to manage trauma-exposed students effectively. Suggestions included expanding family and community engagement, integrating preventive strategies, and providing additional resources for reporting and referral.

Demographic observations showed that female participants (8) highlighted strategies supporting student-centered learning and teacher self-care, whereas male participants (4) emphasized implementation feasibility and integration with school systems. Social workers (3) and psychologists (2) noted the value of ongoing support and monitoring of trauma-exposed children.

## 4. Discussion

This study developed and validated a trauma-informed curriculum for primary school teachers in Limpopo province, addressing critical gaps in teacher preparedness to support learners affected by childhood trauma. The findings highlight a pervasive lack of knowledge and training among educators, consistent with international research showing that teacher understanding of trauma is essential for creating supportive learning environments [45,46]. By providing practical strategies for identifying and responding to trauma, the curriculum equips teachers to address learners’ emotional and educational needs, particularly in under-resourced rural schools where access to mental health support is limited.

Participants found the curriculum relevant, clear, and feasible for implementation, emphasizing the value of experiential learning approaches such as case studies, role-play, and peer feedback. These methods align with previous studies demonstrating that applied, participatory training enhances teacher competence and confidence in implementing trauma-informed practices [47,48]. The curriculum also addresses teacher well-being and self-care, recognizing that managing secondary trauma is essential to prevent burnout and sustain effective classroom practices, a finding supported by research on teacher resilience in high-risk environments [49,50,51].

The curriculum further emphasizes collaboration with mental health professionals and the broader school community, reflecting best practices for holistic, trauma-informed education. By fostering partnerships within schools and linking teachers to external support networks, the intervention promotes a more coordinated and responsive approach to student well-being. These findings have practical implications. Education authorities should consider integrating trauma-informed training into professional development programs, and schools should adopt policies that support both student and teacher mental health.

Implementing such training may enhance classroom safety, foster student resilience, and contribute to broader systemic improvements in educational practice. Future research should also explore long-term impacts and expand implementation to diverse settings, reinforcing the importance of trauma-informed approaches as a cornerstone of inclusive and equitable education in South Africa.

## 5. Limitations

While the development and validation of the trauma-informed curriculum provided valuable insights, several limitations must be acknowledged. First, the study focused on a relatively small sample of teachers (26 participants), which may limit the generalizability of the findings. The participants were primarily from rural communities in Limpopo, and their experiences and perspectives might not reflect those of educators in urban or other geographical settings. Thus, caution should be exercised when extrapolating the results to a broader context.

Moreover, the study was based on qualitative data collected through interviews, which, although rich and detailed, are subject to bias and interpretation. Teachers’ responses may have been influenced by personal experiences, societal norms, or cultural factors that could affect their understanding of childhood trauma and its impact on students. These limitations highlight the need for further studies with larger, more diverse samples and a deeper exploration of how trauma-informed practices can be integrated into various school systems across different regions. While most responses showed high agreement, a few divergent views emerged on family engagement and preventive strategies, highlighting the need for larger, more diverse studies.

## 6. Conclusions and Recommendations

This study successfully developed a conceptual framework and a trauma-informed curriculum tailored for primary school teachers in Limpopo province, South Africa. By integrating empirical insights from teachers, mental health professionals, and existing literature, the curriculum addresses critical gaps in trauma knowledge and equips educators with practical strategies to support trauma-exposed learners. Its implementation has the potential to enhance teacher confidence, foster safer and more inclusive classroom environments, and improve the academic and emotional well-being of children affected by trauma. Furthermore, the framework and curriculum provide a culturally sensitive and resource-conscious foundation that can be adapted to similar rural contexts experiencing high levels of trauma.

Building on the findings and acknowledging the study’s limitations, it is recommended that future research expand to include larger and more diverse samples, incorporating both rural and urban schools to enhance the generalizability of results. Longitudinal studies should also be conducted to evaluate the sustained effectiveness of the curriculum in improving teacher competence and reducing burnout, while simultaneously tracking its impact on learners’ academic performance and emotional resilience. In addition, integrating continuous professional development and fostering partnerships between educators, mental health practitioners, and community stakeholders will ensure that trauma-informed strategies are effectively embedded and maintained across school systems. Such collaborative and contextually grounded approaches are essential to creating lasting, systemic change in addressing childhood trauma within the South African education sector.

Validation results showed strong agreement on curriculum clarity, relevance, feasibility, and teaching methods, with participants highlighting reporting mechanisms, classroom strategies, and professional support. Implementation could boost teacher confidence, create safer classrooms, and improve the well-being of trauma-affected children. Validation results showed strong agreement on curriculum clarity, relevance, feasibility, and teaching methods, with participants highlighting reporting mechanisms, classroom strategies, and professional support. Implementation could boost teacher confidence, create safer classrooms, and improve the well-being of trauma-affected children. The framework and curriculum provide a culturally sensitive and resource-conscious foundation that can be adapted to similar rural settings facing high trauma prevalence.

## Figures and Tables

**Table 1:** Childhood Trauma-Informed Curriculum.

Module	Learning Objectives/ Content	Method of Training	Materials	Method of Assessment	Duration
Childhood Trauma Introduction	To define childhood trauma	Lecturing and having group discussions	Slides and handouts	Short quiz	45 min
	To identify various forms of childhood trauma				
	To identify the effects of childhood trauma				
Recognizing early signs of childhood trauma	Identify the following Behavioral indicators Cognitive signs and Emotional indicators	Role-playing as well as case study analysis	Real-life scenario videos	Participation in role play and short quiz	45 min
Classroom trauma-informed strategies	Techniques to create a safe learning/academic space	Practice exercises and peer feedback sessions	Slides and handouts	Short quiz	45 min
Teachers’ self-care techniques	Techniques to manage secondary trauma effects (self-care routines)	Role-playing and group discussions	Real-life scenario videos	Participation in role play and discussion	45 min
Support systems and collaboration	Identify emergency contact personnel	Lecturing and support group discussions	Slides and handouts	Short quiz	45 min
	Referral processes and community resources				
	Working hand in hand with healthcare professionals				
	Establish a network of support				
Trauma-Informed policies	Integrating practices into the school culture	Lecturing and group discussions	Policy template examples	Policy draft submission	45 min

**Table 2:** Demographic Characteristics.

	Frequency (*n* = 26)	Percent
Age		
20–29	2	7.7
30–39	14	53.8
40–49	6	23.1
50–59	4	15.4
Gender		
Female	14	53.8
Male	12	46.2
Occupation		
Clinical Psychologist	3	11.5
Curriculum Advisor	3	11.5
Primary school teacher	9	34.6
Researcher	4	15.4
Social Worker	7	26.9

**Table 3:** Evaluation scores and average.

Theme	Sub-Theme	Findings (Descriptive)	%
Clarity and Content Relevance	Curriculum relevance	The curriculum adequately addresses essential aspects of early childhood trauma in educational contexts.	92.3%
	Learning objectives	The learning objectives were clearly articulated and achievable.	100%
	Module coverage	The content of each module met the needs of primary school teachers.	96.1%
	Content sufficiency	No additional essential topics were required, indicating comprehensiveness.	57.5%
Teaching Methods and Strategies	Appropriateness of methods	Strategies such as seminars and role-play were suitable for engaging teachers in trauma-informed practices.	96.1%
	Effectiveness of methods	Teaching methods were effective in helping teachers understand and apply trauma-informed practices.	100%
	Sufficiency of methods	No alternative or additional training methods were recommended, reflecting satisfaction.	76.5%
Practical Application and Usability	Feasibility of implementation	Implementation in real school settings was considered feasible.	96.1%
	Resource adequacy	Available resources and materials could adequately support the learning process.	80.8%
	Time allocation	The time allocated to each module was appropriate.	96.2%
	Logistical considerations	No logistical challenges were identified, suggesting smooth integration.	84.4%
Overall Impression	General evaluation	The curriculum received an excellent overall rating.	96.1%
	Suggestions for improvement	No further suggestions for improvement were provided, reflecting overall satisfaction.	76.9%
	Impact potential	The curriculum will enhance teachers’ ability to support and manage trauma-exposed learners more effectively.	95.6%

## Data Availability

The data supporting the findings of this study are not publicly available due to confidentiality and ethical considerations. However, they may be made available by the corresponding author upon reasonable request.
